# Chronic drug treatment among hemodialysis patients: a qualitative study of patients, nursing and medical staff attitudes and approaches

**DOI:** 10.1186/s12882-020-01900-y

**Published:** 2020-06-26

**Authors:** Lee Gilad, Yosef S. Haviv, Inbal Cohen-Glickman, David Chinitz, Matan J. Cohen

**Affiliations:** 1 Faculty of Medicine, Hadassah-Hebrew University Medical Center, Jerusalem, Israel; 2 Department of internal medicine, The Chaim Sheba Medical Center, Ramath-Gan, Israel; 3 Department of Nephrology, Soroka University Medical Centre, Beer-Sheva, Israel; 4 Braun School of Public Health - Department of Health Policy and Management, Hebrew University, Hadassah, Jerusalem, Israel; 5 Clalit Health Services, Jerusalem district, Jerusalem, Israel

**Keywords:** Hemodialysis, Drug adherence, Medical staff, Nursing staff

## Abstract

**Background:**

Dialysis patients have a high pill burden, increasing their care complexity. A previous study in our institution’s dialysis unit found notable discrepancies between medication prescriptions, purchases and patient reports of medication use: overall adherence to medication was 57%, on average; staff reported patients took 3.1 more medication types than actual purchases; concordance of patient purchases and nurse reports was found in 5.7 out of 23.6 months of patient follow-up. We sought to investigate patients and staff concepts and attitudes regarding medication care and to understand better the previously identified inconsistencies.

**Methods:**

We performed a qualitative research based on the grounded theory approach, using semi-structured, in-depth, interviews with patients and staff from the same dialysis unit studied previously, at the Hadassah Medical Center, Jerusalem, Israel.

**Results:**

Though all respondents described a seemingly synchronized system of care, repeated questioning revealed that staff distrust patient medication reports. Patients, on their part, felt that their monitoring and supervision were bothersome and belittling. Along with patients, nurses and physicians, we identified a “fourth” factor, which influences medication care – the laboratory tests. They serve both as biological parameters of health, but also as parameters of patient adherence to the prescribed medication regimens.

**Conclusions:**

Participant responses did not clearly resonate with previous findings from the quantitative study. The central role of laboratory tests should be carefully considered by the staff when interacting with patients. An interaction process, less adversarial, centering on the patient attitudes to medication care, might establish better communication, better cooperation and better patient outcomes.

## Background

Chronic dialysis patients are expected to take multiple medications - on average 17 to 25 pills a day, adding to the complexity of their care [1–4]. There have been pronounced non-adherence estimates, as high as 74% [4–8].

Among patients, adherence with prescribed treatment (dialysis attendance, appropriate nutritional changes and medication adherence) is associated with decreased mortality, [8, 9] and there is evidence that non- adherence to medications is associated with increased healthcare costs and hospitalization [1, 6, 9]. Association between medication non-adherence and undesirable health consequences remains unclear. Assessing this association is challenging due to non-standardized methods defining drug treatment adherence and response [2, 10–15].

Between 2007 and 2009, we performed a study in which we sought to examine and record medication prescription and purchase in a dialysis unit, hoping that identification of inconsistencies could lead to improved care planning. We were surprised by the extent of discrepancies identified and how staff were often misinformed about actual medication use. Overall, there was approximately 57% adherence to medications prescriptions, which varied between medication types. There were medications taken unknowingly to the staff, medications not taken even though staff was under the impression that they were, and dosage discrepancies [16]. Other notable findings included an average discrepancy of 3.1 medication types reported by the nurses but not actually purchased by the patients. We also found that of 23.6 months of follow-up per patient, on average, only in 5.7 months there was complete concordance between actual purchase and nurse reports.

Qualitative research is a strong tool aiding the analysis of socially meaningful behavior, with attention to dynamic aspects of social events and interactions [17]. This methodology can illuminate and identify perception gaps between patients, physicians and nurses and could resonate and elucidate the quantitative results of the latter report [16]. Such a novel approach might assist in any intervention or change in the prescription practices and policy.

In this study we sought to examine, using qualitative methods, patient and staff thoughts and attitudes regarding the role and process of medication use with focus on the previously identified discrepancies.

## Methods

We present a qualitative study in which we interviewed staff and patients in a dialysis unit. We performed semi-structured in-depth interviews, combining open-ended and closed questions. The interview was developd for this study (see supplementary material). The interviews were conducted with eight physicians, 20 nurses and 50 patients. All physicians were internal medicine consultants, two were residents in nephrology (both qualified internal medicine consultants) and six were qualified nephrology consultants. All interviewees were employees and patients in the dialysis units affiliated to the Hadassah Medical Hospital, Jerusalem, Israel, where our previous study was conducted [16].

The study was in accord with the Good Clinical Practice guidelines - it was not interventional, and included only individuals who provided consent. All data was kept anonymous. The institutional review board provided such studies in the dialysis unit exemption from specific approval requests (no coded approvals). There was an interviewee list which was kept during data collection, to ensure participants were not approached more than once. At the end of the data collection process, this list was discarded. There were no exclusions for either staff or patients who consented to be interviewed. All staff were approached and interviewed. Patients were approached based on the convenience of the interviewers. Interviewer visits to the dialysis units were conducted on all weekdays, during daytime between morning and late afternoon. Interviews were not repeated and only one session took place with each interviewee.

The Interviews were conducted by three interviewers, who were clinical pharmacy students who did not know, nor were part of, the staff who treated the patients. The interviewers were not familiar with the previous quantitative study results. The interviewers had a questionnaire with topics alternating between functional topics (medication use, reports) and open-ended questions that allowed the interviewees to elaborate on their perceptions and feelings. Of the research group, only YSH had acquaintance with the patients included in the study. YSH was not involved in facilitating interviews, nor with transcription and saw only anonymized transcribed texts of the interviews. LG, ICG and MJC were and are physicians who do not work in the dialysis unit and DC is a public health scholar. MJC and DC had previous experience in qualitative research; DC published papers and books associated with these studies.

Staff interviews were conducted in the staff room or offices, at times when staff members agreed to meet privately with the interviewer. Patients were approached when attending regular dialysis sessions. They were either interviewed before, during or after the dialysis sessions, at their convenience. Interviews were conducted only in quiet settings, where sufficient sense of privacy was maintained. All interviews were recorded and later transcribed. Transcriptions were not returned to the interviewees for comments, and the patients were not asked for feedback regarding the transcriptions.

The interviews were focused on discrepancies between medication prescriptions and use, and associated communication gaps between patients and staff. We sought to examine whether patients and staff acknowledge these discrepancies and sought to elicit thoughts and feelings about communication and the respective roles each participant had with regard to medical care. The interviewees were told that the purpose of the study was to examine the process of medical care in the dialysis unit.

The analysis was conducted in a systematic research methodology based on the grounded theory approach [18]. In this approach, in contrast to hypothesis testing and induction, themes and categories which emerge from the data are collected to form a proposed theory [17]. The interviews were analyzed in order to identify themes and major categories, while ensuring validity and reliability of the findings. We hypothesized that 50 patients would be sufficient to reach concept and theme saturation. During study analysis all 50 interviews were analyzed and saturation in retrospect was achieved with 20 to 30 participant interviews. As mentioned above, all staff members were interviewed.

**Fig. 1:**
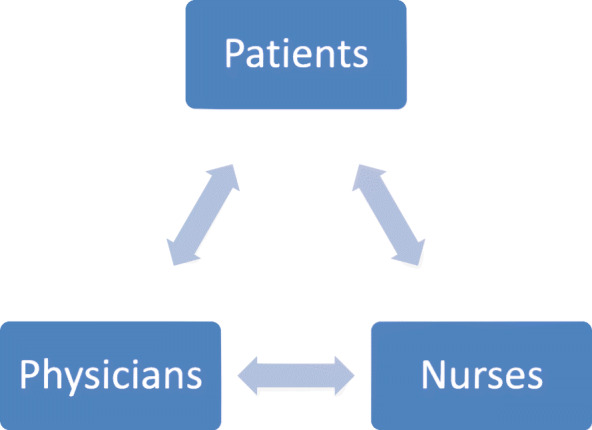
The Initial Framework. When approaching the raw interview transcriptions, it seemed useful to allocate all themes and topics according to the three roles of individuals in the dialysis unit

LG, DC and MJC performed the initial data coding and analysis. In order to interpret the responses, topics were initially designated as either internal (reflecting respondents thoughts or behavior) and external (reflecting how respondents saw others). Initially, we used a framework model (Fig. 1) based on relationships between interviewees – including patients, nurses and physicians. This model was useful to establish a consistent categorization method. During review of the responses, using the “extraction method” (long text formulated into ideas and insights [18–23]) and based on respondents’ descriptions of activities, we generated a revised category scheme mapping dominant elements of content with regard to medication treatment. We did not analyze nor present minor topics, as these were few.

## Results

The ‘**Initial Framework’** depicts a triangular relationship between physicians, nurses and patients regarding medication care (Fig. 1). There is a routine monthly physician visit in which patient medication regimens are reviewed by nurses, presented to the physicians who either confirm the regimens or recommend changes and provide the necessary prescriptions. After reviewing the interviews, we generated a category scheme of the main elements of content regarding medication treatment, which is detailed below.

### The centrality of medications in care

All interviews were focused on medications as the central process of the medical care. Other elements of care, such as food and drink or adherence to dialysis regimens, were not discussed, nor were topics of quality of life, social and familial implications, chronicity, end-of-life decisions and other medical comorbidities.

#### Perceptions of physicians

Medical staff contents were classified according the categories of the “**Initial framework**” – their responses could be easily categorized in accordance with this template. Interestingly, each of these elements was discussed categorically, with little notion of team-work, or mention of none-but-functional patient-nurse interactions.

- Acting as general/family practitioners:

All interviewed physicians raised the view that they are de-facto their patients’ primary-care physicians (family doctors) as well as nephrology consultants. This is a consequence of the repeated monthly visits and the complicated nature of the patients – the nephrologists felt that the community primary-care physicians prefer not to deal with these complicated patients.

*"The GP's feels comfortable leaving the drug issue to the dialysis physician. I think they feel comfortable that we are taking care of the patients*." (*D.2.*)

- Working with the nursing staff:

Delegating responsibilities of treatment emphasis, medication documentation and supervision are the stated roles of the nurses. Physicians noted that they trust nurses and depend on their information.

- Patients:

There are several notions physicians expressed regarding patient role in the process of medical care: partnership, morbidity, neglect/abandonment (purposeful or not), blind trust and despair.

*"I've yet to encounter a patient who argues. I think the more problematic patients usually just nod* and *agree with what comes out of my mouth."* (D.8.).

Physicians highlight adherence to medication regimens prescribed as being key to successful treatment. They describe a variety of strategies they use such as negotiation or medication focusing.

*"…for the coming month - take beta-blockers because your heart rate is high."* (*D.5.)*

However, there were also cases in which the physicians felt they simply didn’t get through to the patients, because of limited patients’ capabilities.

*"There are cases that I'm hopeless and after I've talked with the patient for several times I have him sign a document where it's written he is declining care*" ( D.6.).

Adherence monitoring are at the center of interaction due to fear of sub-optimal adherence and laboratory tests are used to assess the truthfulness of patient reports (see below).

#### Perceptions of nurses

Nurses also related to the classic roles – patients, physicians and nurses. However, there was far more emphasis on relationships and team-work.

*“We are constantly improving quality of life together, and together we have more success” (N.34.)*

Most interviewed nurses considered medication as a pivotal part of care.

*"Medication is the treatment for the disease and, if there is a disease, then we must take care of it" (N.111.*)

Nurses noted and detailed specific elements of their role in the dialysis units, specifically, assessment of adherence.

- Adherence assessment:

The nursing staff consider this their central role in the process of medication care (we did not discuss the dialysis procedures). The interview focused on various elements of this role. Assessment is performed subjectively and objectively. Subjective topics include discussions with patients to evaluate their knowledge of their medications and regimens. Objective elements included vital signs and laboratory test results.

*"I explain why it is important and what are the affects in the short and long term."* (N. 34)

Strategies to confront non- adherence included rational explanations, emotional support, motivational tactics, direct problem solving (giving the medication after the dialysis session, organizing the pills), memory checks and cognitive confirmations. With some patients, “passive” guidance is the communication strategy, allowing the patients to raise the medication adherence topics rather than imposing the issue on them:

*"Every time they come we ask them how they feel…(and this leads them to talk about medications)."(N.42)*

- Relationship with physicians:

Nurses reported that often physicians consult with nurses before summarizing monthly visits. However, physicians are considered the case managers and the higher authority. Due to comorbidities, patients sometimes require or receive consultation from non-nephrology experts. In such cases, nurses notify the nephrologists accordingly.

- Physician-patients relationships:

Almost all nurses mentioned the centrality of the relationship between patients and physicians. Most feel that patients see the nurses as assisting supervisors.

*"Patients see physicians as experts, and they see nurses as ones who supervise the medication plan." (N.39.)*

#### Perceptions of patients

The patient interviews often revealed seemingly contradictory elements and feelings. Though highly respectful of the professional staff, patients also reported that the adherence monitoring was sometimes a nuisance. Similarly, while answering that medications and regimen adherence are important, complex regimens and perceived side-effects can be associated with sub-optimal adherence. Overall 48 (96%) patients agreed that medical treatment was important, 46 (72%) reported that they were not the person who went to the pharmacy to purchase the prescribed medications, 46 (92%) stated that they were truthful in their reports to the staff about medication adherence, 32 (64%) reported using a pill-box to help take the medications in an orderly way, 41 (82%) agreed that physical well-being is associated with medication adherence and 45 (90%) responded that they trusted the staff in the dialysis unit.

- Medical and nursing staff:

The interviewed patients’ description of the roles of nursing and medical staff was consistent with the “Initial model” and the responses of the professional staff, as presented above. They hold the physicians with the highest esteem and initially replied that they (the patients) are seldom inaccurate when reporting medication adherence.

- Medication reports:

Most patients reported being truthful in their reports to the staff about mediation adherence. Yet, on repeated questioning, discrepancies were sometimes reported.

*“I take all medications prescribed……I would never give an inaccurate report of what I take……” (*P. 77)

*“There was this time I forgot to take medication…I was away….(in response to a question about occasions where medication non-adherence was not reported)”* (P. 77).

One had mentioned being told-off for adherence issues. Most mentioned that the staff seems dissatisfied with them (the patients) when laboratory results were abnormal. To some, it seemed that there is a direct link between laboratory results and medication changes.

*“Once a month the nurse and physicians review the lab tests and only then decisions regarding medications are provided to me.”* (P. 85).

#### Laboratory tests

Laboratory tests were an important part of the medical follow-up. Physicians made frequent use of laboratory tests to follow patient’s physiological condition, and when necessary, modifying the treatment program. Laboratory tests were seen by physicians as a significant tool reflecting patients’ true medical condition.

*"It's like a polygraph here; I can see he did not take his medication"*. (D.3)

"*If the tests are OK, then I do not care what he says he takes*." (D.6)

Nursing staff saw the laboratory test results as important for the physicians and as a tool to help assess the truthfulness of the patients’ reports. Nurses referred to laboratory tests as a monitoring checkpoint. In addition, nurses used laboratory tests as a “warning signal” of sub-optimal adherence, as do physicians.

"*We keep track of their tests, so we have clues as to what the patients take*". (*N.34*)

"*We try to teach the patients about their medications effects. We print the blood tests and we show them their high phosphorus, for example* "(N.55)

Among 50 patients, only 11 patients referred to laboratory tests. Understanding the nature of laboratory tests within the continuity of care had special importance for those patients. Some of them even saw good results as rewarding.

"*The nurse and the physician show us the results, and how we're progressing" (P.96)*

### Analysis and model

Patients and staff all provided responses which portray a relatively synchronized and harmonious system. This is in contrast to our findings in the previous quantitative study, where only about half of medications were taken in accordance with medical instructions. Additionally, in the previous study, we found that the nursing and medical staff were not informed of these discrepancies [16]. In the analysis of the interview, we did identify two parallel and contrasting narratives. The first - an overt notion that “the system works” – patients are committed to their own well-being and trust the staff to instruct them and, thereby, lengthen their life and improve its quality. Staff perform their duties and provide professional care and support. The second narrative – in which patients need to be constantly monitored and the staff has to be alert regarding their adherence. In this narrative the staff is vigilant and uses laboratory tests to guide care and assess adherence. Patients expressed discomfort with these paternalistic aspects, which they feel are demeaning and a nuisance.

**Fig. 2:**
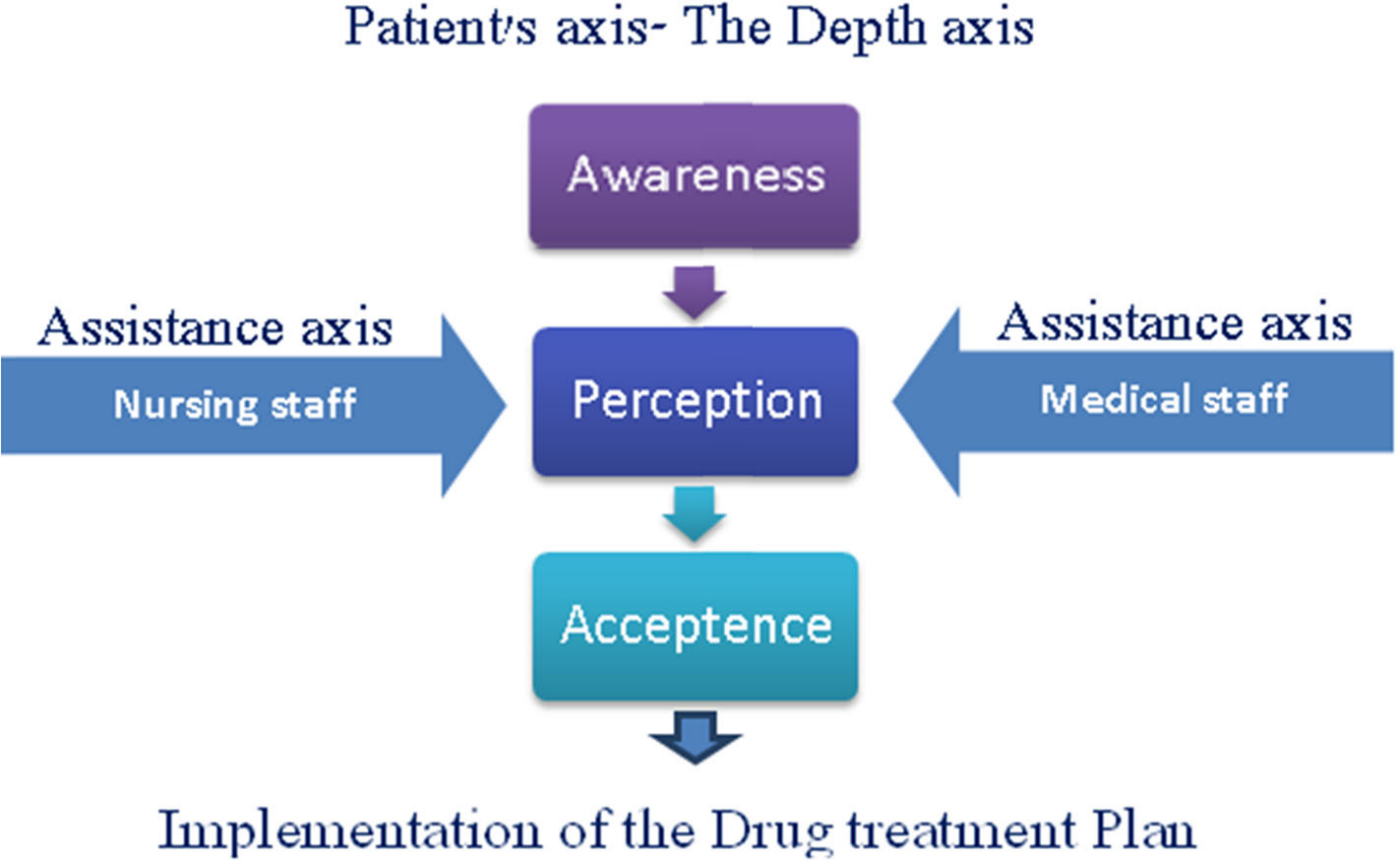
Proposed model. This proposed model centers on the patients, and instead of designating them as either adherent or non-adherent, the focus is their attitude towards medical care. There is a hierarchical element in which the patients move from one milestone to the next

These findings could represent the significant discrepancies and communication breakdown demonstrated in our previous study [16].. Rather than a triangular model (Fig. 1), we feel that a revised model of care could enable a less adversarial and less paternalistic process (Fig. 2). The model centers on the patients and consists of three milestones: ‘awareness’, ‘perception’ and ‘acceptance’. Staff members draw their identity as facilitators improving patient welfare. Laboratory test results function as a prism through which the participants are assessed medically and are assisted in the process of care.

#### The awareness milestone

Patient understanding and knowledge of the medications, which they are prescribed and expected to take, varies. Awareness is determined by the importance patients allocate to drug therapy, which can be appreciated by the methods used by patients to take medicines, such as pill boxes, reminders, notes etc.

#### The perception milestone

Patient perceptions regarding the essence of medical treatment and how it enables them to benefit from the continuity of care. The concept varies according to the level of understanding of their medical condition. Most patients indicated that they understand the importance of medical care. Yet, most of them report that they often forget taking medicines. This demonstrated a perceptual gap – though patients believe drug treatment is important – they do not devise successful adherence strategies.

#### The acceptance milestone

The patients are the regimen gatekeeper and their role keeps them involved in their care. Acceptance is reached when a patient associates the importance of drug treatment with their medical condition and their well-being. Patients, at this stage, are in control. They make their own decisions, sometimes in contrast to the medical staff’s recommendations.

#### Assistance Axis- medical staff

Physicians are perceived by patients to be the highest professional authority. Physicians serve both as professional consultants and de-facto primary care physicians, given the frequent interaction with the patients. This dual function often forges deep and meaningful relationships between physicians and patients. However, it should be noted, most physicians were critical of patients’ reports, as they suspected patients wish to satisfy them. As a result, many physicians found it difficult to believe patients take drugs according to their treatment plan. In turn, this approach towards patients, along with a close relationship they have with them, can be perceived negatively by patients. Many patients felt dissatisfaction with the fact that physicians recurrently asked them about medication adherence.

Assessing the patients’ milestone and appreciating that both cognitive and emotional elements influence their adherence can enable a more compassionate and accepting work process, adapting communication and recommendations with respect to the patients’ state of mind.

#### Assistance Axis- nursing staff

Physicians and patients consider the nurses mostly in a functional role, who assist physicians “who simply don’t have the time for all the necessary communication”. In contrast, most nurses consider themselves as a pivotal part in the care-team. Still, there are nurses who described an important yet passive role, providing a link between patients and physicians; while others see their role as more active, suggesting changes, interventions and helping physicians and patient reach, what they feel, are better decisions.

## Discussion

We found that while patients, nurses and physicians describe a process of care in which trust and cooperation exist, the issue of adherence monitoring generates a persistent undertone, which is associated with contrary feelings of mistrust and annoyance. The laboratory test results serve as an additional party to these interactions. The staff consider the lab results as objective parameters in order to keep patients in line.

Given the findings of our previous study and the parallel narratives which arise from the interviews, we believe that a model of interactions between the three parties does not provide a sufficient framework to understand the interactions and attitudes described in this study. Additionally, the inconsistencies found in the previous study are somewhat alien to the tri-party initial framework.

We propose a model, centered on the patients, where the staff can set aside confrontation, associated with adherence monitoring, and provide support with respect to the patients’ attitude towards their health/morbidity status and medications. The model incorporates previous findings of qualitative and quantitative studies examining low adherence among hemodialysis patients.

Standard methods to evaluate adherence have not been set or widely accepted [9, 10, 14, 24]. There have been specific assessments of the adherence to phosphate binders. Focusing on these medications has the advantage of the ability to assess response with laboratory tests, resonating our findings of their ‘role’ in care. Others have shown that an interplay between patient knowledge, belief and attitude greatly impacts their adherence. Simply following up serum calcium and phosphate levels and increasing dosage did not improve test results, nor improve adherence [2, 25, 26].

Allen et al. describe a longitudinal qualitative study of patient attitudes in a dialysis units, where, over 2 years of observations and interviews, they identified the existence of adversarial relationships between staff and patients, uncovering lack of trust between the parties. Similar to our model, the authors proposed that the staff appreciate the patients’ whole complexity, including perception and attitudes, as a strategy to improve interactions and patient well-being [27]. Indeed, a systematic review of qualitative studies, assessing the life experiences of patients with chronic kidney disease, found consistent reports of patients burdened by the disease and also by the treatment and system which aims to lengthen and improve their lives [28].

The concept of patient milestones along the patient therapeutic process was proposed by Curtin et al. who showed that adherence to treatment decreased with time and medications that facilitated immediate relief of symptoms had higher adherence. Patient experiences were categorized and a process of “restructuring of the self” was identified, in which patients adapt to their evolving reality [29, 30]. Gregory et al. also found that a process of change in self-identity plays a crucial role in patient behavior [31]. These and similar themes were identified in a review of patient experiences while in dialysis care [32]. Lower adherence has been shown to be associated with issues of acceptance of the disease status, especially among people shortly after initiation of dialysis sessions. Acceptance of the circumstances was considered to be associated with better adherence [33, 34]. Patients’ attitudes towards changes in their lives can impact their adherence preferences. Some might be more inclined to forgo interventions which, they feel, are constricting their ability to maintain their previous lifestyle [35] [36, 37]..

We feel the proposed model could also be beneficial to the staff, who seem to rely on test results to assess patient adherence. Abnormal test results could suggest that disease progression is at play, notwithstanding adherence with staff recommendations. Another option, is that the patient’s assessment did not pick up on all aspects which required attention, and the patient’s lack of improvement / deterioration results from less-than-optimal recommendations. Taking a step back from the authoritative role could inject a more humble approach and provide better care in a less stressful environment.

Our findings did not resonate our previous quantitative results, which demonstrated significant discrepancies between patients medication purchasing and reports to the staff [16]. We did not repeat the methods from the previous study in which we compared patients’ specific purchasing data with reports to the staff. We preferred to avoid these potentially confrontational interactions with patients. We do not believe that we would have elicited ‘more truthful’ answers, and fear that compliance towards and during the interview would have decreased. Our findings might seem limited, as this is a single center study. Additionally, we did not collect any personal characteristics from respondents nor from patients who declined, do we have any ability to assess selection bias. The response rates were high, and few patients declined our invitation to talk. However, most of our findings were consistent with the known body of research assessing the process of care and adherence in dialysis patients. Additionally, our prior knowledge of the actual medication patterns, prescription and purchases allowed a more realistic analysis of the qualitative data. Finally, we were able to include all working physicians, most of the nurses and a large number of patients, providing robust data from which we could draw conclusions.

Framing a different model and working with it requires a change of thought on the part of the staff. It is not simply being more emphatic, as it requires that staff see themselves as serving an autonomous patient. Autonomous to decide, feel and fail. Rather than correcting and keeping the patients on a tight leash, the model defines patients as central, with staff serving the patients as best as they can, appreciating that there is an evolution of attitudes and perceptions in the patients’ process of coping with their disease.

## Conclusions

We found that laboratory test results serve a key function, not only from a physiological sense, but as an indicator of patient adherence. While overt responses suggested that medication care and communication between staff and patients is coordinated, there was also content which suggests that there is staff distrust regarding patient adherence and truthful report of medication behavior. Patients felt uneasy about the scrutiny they were under. We propose a model which focuses on the patients’ attitude towards medication care. We believe that the model can serve to harmonize the management and follow-up of medication prescription and use. Appreciation of patients’ attitude can direct the focus of their needs and create a less adversarial environment, where all members work together to enable shared decision making, thereby improving communication and patient outcomes.

## Supplementary information

**Additional file 1.** Patient interview.

## Data Availability

The datasets generated and/or analysed during the current study are not publicly available - the transcribed texts are in Hebrew. They were translated to English for the purpose of this publication but are available from the corresponding author on reasonable request

## Abbreviations

USRDS: The United States Renal Data System
IRB: The institutional review board
D: Doctor
N: Nurse
P: Patient
GP: General Physician
