# Supplementary material for: Chronic drug treatment among hemodialysis patients: a qualitative study of patients, nursing and medical staff attitudes and approaches
Source: BMC Nephrol. 2020 Jun 26;21:239. doi: 10.1186/s12882-020-01900-y (PMC7318765; doi:10.1186/s12882-020-01900-y)
Supplement: Supplementary file 1 — Additional file 1. Patient interview. [file 12882_2020_1900_MOESM1_ESM.docx]

**Patient interview**

What importance do you feel medication care has for you? Why?

How often do you go to the pharmacy?

Does anyone collect/purchase your medications for you?

Do you collect/purchase medications for anyone other than yourself?

Who provides you with prescriptions?

Do you report your medication regimens to the nursing staff in the dialysis unit?

Do you report medications that are not associated with your kidney disease and care in the dialysis unit?

Have you ever knowingly provided untruthful / inaccurate information regarding medication care?

Have you ever reported taking a medication, which you did not really take?

Have there been occurrences in which you were advised to stop taking a mediation and you continued to take it?

How do you organize your medications at home? How do you remember which medication to take at which time?

Have there been occasions where you were told off or felt negative feedback due to adherence issues with medications? Do you know if other patients had such experiences?

Is there an association between adherence to medication care and wellbeing or being admitted to hospital care?

Are there medications you know make you feel better while the physicians think otherwise?

Are there medications you know make you feel worse while the physicians think otherwise?

Do you ever feel than you know better than the physicians with regard to management of your medication care?

**Nurse interview**

What importance do you feel medication care has for patients? Why?

How do you receive reports from the patients regarding the mediations they take?

Does a nurse review medication care on each patient visit?

Do you ask to see the medications? Do you rely of patient report and memory?

Do some nurses fill medication reports without discussing the topic with patients?

How do the physicians communicate their recommendations to the patients?

How do you know which medication the patients are supposed to take?

What do you do when you notice that patients do not take medications they are supposed to take?

Do you think the patients are reliable in their reports? If not – why? When?

Do you criticize patients who are not adherent to mediation care?

Do physicians criticize patients that are not adherent to mediation care?

Do patients with better adherence receive better care?

Do patients with better adherence receive nicer/better attention?

Are there cases in which patients know better than staff about what is good for them?

Has it occurred that physicians provided recommendations that you did not agree with? Did you communicate that to the patients?

**Physician interview**

What do you talk to patients about during follow-up at the dialysis unit?

Do you find yourself dealing with medical issues not directly related to the dialysis and chronic kidney disease care?

What importance do you think medication care has for your dialysis patients?

Do you ask your patients which medications they take?

How do you know which medications your patients take?

Do the nurses provide reports of patient medication care?

Is the information you are provided with accurate? Why are there inaccuracies?

Which medications do you focus on?

Are you interested in all the medications the patients take or only those relate to dialysis and chronic kidney disease?

Do you provide prescriptions that are not related to dialysis and chronic kidney disease care?

Do you make recommendation of medication change in matters no associated with dialysis and chronic kidney disease care?

When you feel that medication care need not be changed – do you actively communicate that or avoid recommending any change?

When you recommend change – how do you do that? What actions are involved in order to create the change?

Do you communicate with the patient’s family physician?

In your opinion, to what extent do you fell patients trust your recommendations?

Are there cases in which patients know better than staff about what is good for them?

Have you had disagreements with patients regarding medical care?

Does poor adherence lead to patient admission to hospital?

Do you discuss medication adherence with patients? Do you discuss inaccuracies in patient reports?

Have you reduced regimens or stopped medications in order to improve adherence?
